# Modulating Observation-Execution-Related Motor Cortex Activity by Cathodal Transcranial Direct Current Stimulation

**DOI:** 10.3390/brainsci9050121

**Published:** 2019-05-26

**Authors:** Fengxue Qi, Michael A. Nitsche, Volker R. Zschorlich

**Affiliations:** 1 Department of Movement Science, Faculty of Philosophy, University of Rostock, 18057 Rostock, Germany; volker.zschorlich@uni-rostock.de; 2 Department of Psychology and Neurosciences, Leibniz Research Centre for Working Environment and Human Factors, 44139 Dortmund, Germany; nitsche@ifado.de; 3 Department of Sport Training, Sport Coaching College, Beijing Sport University, Beijing 100084, China; 4 Department of Neurology, University Medical Hospital Bergmannsheil, 44789 Bochum, Germany; 5 Faculty of Medicine, University of Rostock, 18055 Rostock, Germany; 6 Department Ageing of Individuals and Society, Faculty of Interdisciplinary Research, University of Rostock, 18051 Rostock, Germany

**Keywords:** movement observation, movement execution, transcranial direct current stimulation, motor cortex activity

## Abstract

The aim of this randomized sham-controlled study was to examine the impact of cathodal transcranial direct current stimulation (ctDCS) of the primary motor cortex (M1) during movement observation on subsequent execution-related motor cortex activity. Thirty healthy participants received sham or real ctDCS (1 mA) over the left M1 for 10 minutes, respectively. The participants observed a video showing repeated button pressing tasks of the right hand during the sham or real ctDCS, followed by performance of these tasks by the right hand. Motor-evoked potentials (MEP) were recorded from the resting right first dorsal interosseous muscle before movement observation during the sham or real ctDCS, immediately after observation of actions, and after subsequent movement execution. The results of the ANOVA showed a significant main effect on the group (*F*_1,28_ = 4.60, *p* = 0.041) and a significant interaction between time and the group (*F*_2,56_ = 5.34, *p* = 0.008). As revealed by respective post hoc tests, ctDCS induced a significant reduction of MEP amplitudes in connection with movement observation (*p* = 0.026, Cohen’s *d* = 0.861) and after subsequent movement execution (*p* = 0.018, Cohen’s *d =* 0.914) in comparison with the sham stimulation. It is concluded that ctDCS during movement observation was effective in terms of modulating motor cortex excitability. Moreover, it subsequently influenced execution-related motor cortex activity. This indicates a possible application for rehabilitative treatment in syndromes with pathologically enhanced cortical activity.

## 1. Introduction

Movement observation or execution-related cortical networks are activated when individuals are conducting an action or observe the identical movement performed by another individual. These networks include the primary somatosensory cortex, primary motor cortex (M1), ventral premotor cortex and inferior frontal gyrus [1,2]. Alpha and beta frequency oscillations desynchronize within these networks during action observation and execution [3,4,5]. These alpha (8–13 Hz) and beta (14–30 Hz) oscillations in the electroencephalogram (EEG) are referred to as mu and beta rhythms and reflect mirror neuron activity [5,6,7]. 

Modulation of the activity of respective networks via non-invasive brain stimulation might alter respective task-related physiological processes and ultimately affect performance. Transcranial electrical stimulation is suitable to modulate cortical excitability and activity by a weak electrical current, which modulates synaptic activity and/or neuronal resting membrane potentials [8,9]. Cathodal transcranial direct current stimulation (ctDCS) results in long-term depression-like effects [8,10]. The effects of transcranial direct current stimulation (tDCS) on motor cortex activity are dependent on the state of the stimulated cortical region before and/or during the application [9]. With respect to treatment of brain diseases associated with pathological enhancement of cortical activity or excitability via cathodal tDCS, some studies have reported that ctDCS applied over an epileptogenic focus reduces epileptic EEG abnormalities [11,12] and seizure frequency in epilepsy [13]. Clinical studies moreover showed that cathodal tDCS over the contralesional M1 combined with motor training rebalances bi-hemispheric activity and facilitates motor performance in stroke patients [14,15] and in children with cerebral palsy [16,17]. Furuya et al. describe that ctDCS applied over the affected motor cortex in musicians cramp, combined with anodal tDCS over the contralateral motor cortex and bimanual finger movements, reduces dystonic symptoms in these patients, most probably due to a reduction of pathologically enhanced activity of the affected M1 [18]. 

In this study, we contrasted the effects of ctDCS and sham stimulation application to M1 during movement observation on subsequent execution-related motor cortex activity. It was expected that cathodal tDCS would reduce movement observation-related motor cortex activity, and that it subsequently would reduce execution-related motor cortex activity, as compared to a sham stimulation.

## 2. Materials and Methods

### 2.1. Participants

Thirty healthy adults (mean age, 25.93 ± 4.75 years; 12 females) participated in this study and were randomized to receive sham or real cathodal tDCS. They gave written informed consent. The ethics committee of the University Medicine Rostock approved this study and the study meets the standards of the Declaration of Helsinki. None of the participants had pregnancy, family history of epilepsy, the presence of neurological, psychiatric or musculoskeletal disorders, central nervous system acting medications, major medical diseases, cardiac pacemakers, skin lesions near the region of stimulation, or metal implants. They had normal or corrected-to-normal vision. The Oldfield’s Edinburgh Handedness Inventory [19] was used to evaluate handedness, and all participants were right-handed. Each stimulation condition was performed in 15 participants.

### 2.2. Monitoring of Motor Cortex Excitability

A single-pulse transcranial magnetic stimulation (TMS) over the left M1 representation of the relaxed right resting first dorsal interosseus (FDI) muscle induced motor-evoked potentials (MEP) which were recorded to monitor motor cortex excitability changes. TMS with a biphasic pulse was conducted by a MagPro R100 magnetic stimulator (Medtronic, Skovlunde, Denmark) with a D-B80 coil. In order to minimize head to coil movement, a chin-forehead rest was used to stabilize the head of the participants. The magnetic coil was held tangentially to the skull over the left M1. A mechanical arm (Manfrotto Feltre, Italy) fixed the handle pointing backwards and laterally 45° from the midline. The optimal magnetic coil position (hotspot) was determined by a moderate suprathreshold stimulation intensity to constantly elicit the largest MEP in the right resting FDI muscle. The intensity of TMS was determined as the percentage of maximal stimulator output (% MSO) which elicited peak-to-peak MEP amplitudes of approximately 1 mV (SI1mV) at baseline before the intervention. This intensity was kept constant for the remaining experiment. Twenty MEPs were obtained from the right FDI muscle for each time bin using a pair of Ag-AgCl cup electrodes with a surface area of 3 mm^2^ (GE Medical Systems, Milwaukee, USA) in a belly-tendon montage. The ground electrode was positioned over the right lateral biceps brachii muscle. The electromyographic (EMG) signals were amplified (input resistance of 10 GΩ, bandwidth of 1–1000 Hz, Biovision, Wehrheim, Germany) with an amplification rate of 1000. DIAdem software was used to process EMG signals. 

### 2.3. tDCS

Cathodal tDCS (1 mA) was delivered for 10 minutes (5 s of ramp-up and ramp-down) by a battery-driven electrical stimulator (BrainSTIM, EMS, Bologna, Italy) and applied through a pair of 25 cm^2^ surface saline-soaked sponge electrodes. The cathode electrode was placed over the defined left M1 ‘hotspot’ and the anode electrode was positioned over the right supra-orbital area. The direct current was switched on for 30 s and then turned off during sham stimulation.

### 2.4. Movement Observation and Execution

The participants kept their hands in a relaxed position. They comfortably seated in front of a computer screen (24-inch) to watch one movement observation video, which displayed the right hand pressing buttons. The video was 10 minutes long and incorporated 20 short clips. Twenty-second long clips in natural speed were presented 10 times. At half of the natural speed, 40 s long clips were presented 10 times. At the beginning, the 20 s long clip was displayed, followed by a 40 s long clip. Low-speed movements were included because these result in a more prominent modulation of motor cortex activity [20]. The clips showed that whenever a black spot changed into a red spot in a regular sequence, a human hand reached for the appropriate round button immediately, pressed it with the index finger only, and quickly got back to the original position afterwards. Participants were instructed to concentrate on the performing finger and the button press task. They were instructed to count the number of button presses, because attention is an important mediator of performance, and performance-related physiological effects [21]. 

After movement observation, participants performed a movement execution task (160 s) which was identical to the observed video. A 24-inch computer screen and a custom-made button-box with 4 red round buttons were positioned on the table in front of the participant. The distance was 20 cm from the right hand to the button-box. The four buttons corresponded to the black spots shown on the computer screen. When a black spot changed into a red spot in a modeled response sequence on the computer screen (each 3 s), participants pressed the button with the right index finger immediately, and quickly moved back to the initial hand position afterwards. The left hand always remained in the resting position.

### 2.5. Experimental Procedures

**Figure 1:**
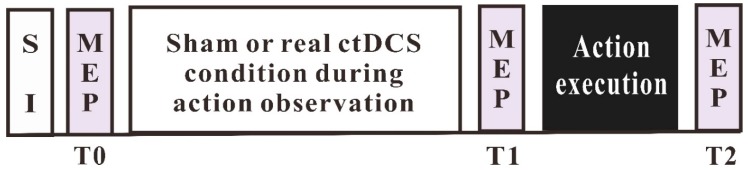
Timeline of the experimental protocol.

The participants received either ctDCS or a sham stimulation, while watching the movement observation video. Movement execution was conducted after the termination of action observation and tDCS. MEPs were recorded before (T0) and after (T1) movement observation combined with the sham or real ctDCS, and then after movement execution (T2). For an overview of the experimental procedure, refer to Figure 1.

### 2.6. Statistical Analysis

SPSS (version 22.0; IBM, Armonk, NY, USA) and Prism (Version 8; GraphPad Software Inc., San Diego, CA, USA) were used to perform statistical analyses. Normal distribution of the data was confirmed by Kolmogorov-Smirnov tests. Independent-samples t-tests were used to examine differences of demographic and physiological data between experimental conditions respectively, including age, SI1mV, and baseline measurements of MEP amplitudes. Gender distribution differences between groups were examined via a χ^2^ test.

For MEP analysis, first, the mean values of the peak-to-peak amplitudes of 20 MEPs for each time bin were calculated individually. If EMG activity larger than 50 μV was present in the 300 ms time-window before TMS, the respective MEP was excluded. A repeated measures ANOVA was performed to analyze MEP amplitude data with the between-subject factors tDCS (sham and real ctDCS condition), and Time course. The assumption of sphericity was examined by Mauchly’s test and the Greenhouse-Geisser correction was applied if necessary. Fisher’s post hoc tests were performed to determine differences between groups. Cohen’s d was used to calculate effect sizes. A significance level of *p* < 0.05 was used for all statistical tests.

## 3. Results

**Figure 2:**
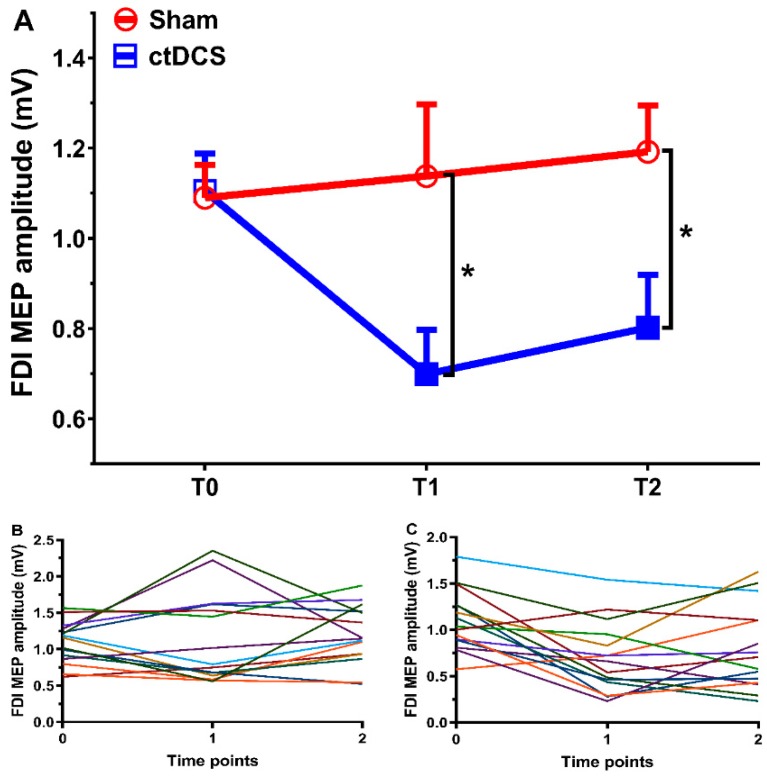
Results of the study. For time points T0, T1, and T2, averaged MEP amplitudes of the first dorsal interosseus (FDI) muscle for all participants (**A**) and individual MEP amplitude in the sham (**B**) and cathodal transcranial direct current stimulation (ctDCS) (**C**) groups are depicted. Filled symbols indicate significant differences of MEP amplitudes after movement observation and execution in comparison to baseline values. Error bars represent standard error of means. * denotes significant differences between groups at *p* < 0.05.

All participants tolerated ctDCS well. There were not significant differences with respect to age, gender, SI1mV, and baseline MEP amplitudes between groups (all values of *p* ≥ 0.136; Table 1). The results revealed a significant main effect for the group (*F*_1,28_ = 4.60, *p* = 0.041) and interaction between time and the group (*F*_2,56_ = 5.34, *p* = 0.008), but the main effect of time was not significant (*F*_2,56_ = 2.76, *p* = 0.072). The post hoc tests showed no significant differences between all time points in the sham stimulation condition. MEP amplitudes were however significantly decreased following ctDCS in comparison with the sham stimulation at time point T1 (after movement observation) (*p* = 0.026, Cohen’s *d =* 0.861) and T2 (after movement execution) (*p* = 0.018, Cohen’s *d* = 0.914, Figure 2A). In comparison to the respective baseline values, there were significant differences of MEP amplitudes from T0 to T1 (*p* = 0.001, Cohen’s *d* = 1.156) and T0 to T2 (*p* = 0.005, Cohen’s *d* = 0.772) in the real ctDCS group. In the real ctDCS group, 13 of 15 (86.67 %) participants at T1, and 11 of 15 (73.33 %) participants at T2 had reduced MEP amplitudes in contrast to the baseline (T0) (see Figure 2C).

SI1mV (% MSO) refers to the intensity of TMS required to elicit an average motor evoked potential (MEP) of approximately 1 mV. ‘-‘ refers to no data available. The mean values ± standard deviations are shown for the sham and real cathodal tDCS sessions. 

## 4. Discussion

In the sham stimulation condition, there were not significant changes in MEP amplitudes at all time points. Thus, in accordance with another study with the same task, movement observation followed by movement execution did not promote MEP amplitude alterations when no effective stimulation was applied [22]. This result is also compatible with other studies in the field. Movement observation alone did not enhance contralateral motor cortex excitability immediately after movement observation [23,24]. Cortical activity returned to baseline levels within a few seconds [25], as shown by direct recordings of mirror neurons in monkeys [26,27] and indirect measures of neural activity in human motor regions [28]. 

Cathodal tDCS applied during movement observation induced a reduction of MEP amplitudes, and this effect remained after movement execution in comparison to the sham stimulation condition. Thus ctDCS had antagonistic effects on the modulation of motor cortex activity which was not abolished by movement observation or execution. This might have clinical implications, namely when symptoms are caused by task-related over-activity or lesions of respective cortical regions. Recent clinical studies have shown that ctDCS over the left sensorimotor cortex combined with action observation and electromyographic biofeedback training improved dystonic posture and writing movements of the right upper limb in a patient with writer’s cramp [29]. Re-training of finger movements in musician’s cramp [18] was improved by cathodal tDCS over the respective motor cortex. Cathodal tDCS combined with movement observation and/ or training thus might improve abnormal cortical excitability and enable a re-arrangement of motor representations. It was also reported that cathodal tDCS over the non-lesional M1 combined with movement execution facilitates motor performance in stroke patients [14,15] and in children with unilateral cerebral palsy [16,17]. The suggested mechanism is that ctDCS coupled with motor training downregulates activity of the non-lesioned hemisphere, while it upregulates activity of the lesioned hemisphere. The latter is via movement execution and reduced transcallosal inhibition, and thus re-balances bi-hemispheric activity and improves sensorimotor representations. In most motor performance studies, anodal tDCS enhanced cortical excitability and improved motor performance [30,31]. This is thought to be caused by improved induction of task-related LTP [32,33]. It was however also shown that cathodal tDCS can improve motor performance under certain conditions [34,35]. This could be due to a signal-to-noise-enhancing effect of the stimulation, which might be beneficial for noisy tasks [36], or when non-selective hyperactivity of a task-relevant area otherwise would reduce performance. This is assumed to be the case in occupation-related dystonia [18].

In the ctDCS group, 86.67 % of the participants at T1 and 73.33 % of the participants at T2 had reduced MEP amplitudes in comparison with the baseline. Given former descriptions of high variability of cathodal tDCS effects [37,38], which might be partially driven by relatively complex non-linear effects of this stimulation protocol [39,40], these effects are remarkably stable. One reason for this stability in the present study might be the known state-dependent effect of tDCS [9]. Brain states might be more variable under resting conditions, which is the context of most studies showing high variability. In the present study, brain states with respect to the target area might have been relatively stable because of task performance during stimulation.

The limitations of this study include the lack of electroencephalographic recordings and motor performance data (technical limitation of the custom-made button-box), which would have improved mechanistical understanding, and evaluation of behavioral consequences. In addition, this pilot study did not include a ctDCS condition alone, and thus information on how ctDCS interacted specifically with movement observation is not available. The aim of this study was not to induce effects of maximum size, but to deliver proof of principle data. Future studies might show if recently developed optimized ctDCS protocols deliver larger effects [40]. 

## 5. Conclusions

It was demonstrated that ctDCS during movement observation was effective in terms of modulating motor cortex activity. Moreover, it subsequently influenced execution-related motor cortex activity. This evidence might guide the implementation of ctDCS combined with task performance to enhance physical therapy in clinical practice in syndromes with pathologically enhanced cortical activity.

## Figures and Tables

**Table 1:** Demographic information, SI1mV, and baseline measurements of motor-evoked potentials (MEP) amplitudes (mV) of the intervention groups.

Group	*n*	Gender (F/M)	Age (Years)	SI1mV	MEP (Baseline)
Sham	15	4/11	26.13 ± 3.98	44.13 ± 10.45	1.09 ± 0.28
Real	15	8/7	25.73 ± 5.55	45.67 ± 11.25	1.11 ± 0.32
Between group	-	*p* = 0.136	*p* = 0.822	*p* = 0.702	*p* = 0.888
